# P-1493. Public Health Impact and Cost-Effectiveness of Vaccination Strategies Against Invasive Meningococcal Disease in Adolescents in the United States

**DOI:** 10.1093/ofid/ofaf695.1677

**Published:** 2026-01-11

**Authors:** Oscar Herrera-Restrepo, Hiral Shah, Ginita Jutlla, Jonathan Graham, Mei Grace, Justin Carrico, Zeki Kocaata, Sara Poston, Diana Clements, Anar Andani, Cindy Burman

**Affiliations:** GSK, Philadelphia, PA; GSK, Philadelphia, PA; GSK, Philadelphia, PA; RTI, Research Triangle Park, North Carolina; RTI, Research Triangle Park, North Carolina; GSK, Philadelphia, PA; GSK, Philadelphia, PA; GSK, Philadelphia, PA; GSK, Philadelphia, PA; GSK, Philadelphia, PA; GSK, Philadelphia, PA

## Abstract

**Background:**

Vaccination against invasive meningococcal disease (IMD) caused by serogroups A, B, C, W, and Y in United States (US) adolescents consists of two schedules: Standard of care (SOC): Q-Q-B-B (quadrivalent MenACWY [Q] routine at 11 and 16 years [y]; MenB [B] under shared clinical decision-making [SCDM] at 16 y and B 6 months [m] later) or SOC: Q-P-B (Q routine at 11 and 16 y; pentavalent MenABCWY [P] under SCDM at 16 y and B 6 m later). We estimated how public health impact (PHI) and cost-effectiveness (CE) of IMD vaccination would be affected by revisions to the adolescent meningococcal vaccine schedule proposed by the Advisory Committee on Immunization Practices (ACIP) in June 2024.

**Table 1.**
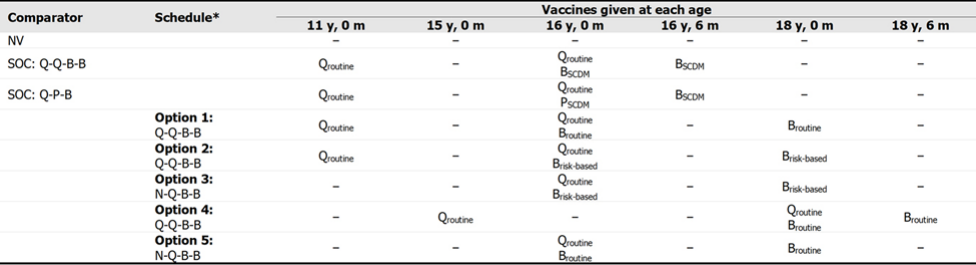
Schedule options discussed at the June 2024 ACIP meeting and evaluated in the model for the adolescent schedule revision ACIP, Advisory Committee on Immunization Practices; B, MenB vaccine; IMD, invasive meningococcal disease; m, months; N, no vaccine given; NV, no vaccination; P, MenABCWY vaccine; Q, MenACWY vaccine; SCDM, shared clinical decision-making; SOC, standard of care; y, years; –, no vaccine given at age. Routine refers to routine vaccine recommendation. Risk based refers to vaccine recommendation for only those who are at higher risk of IMD (e.g., college students). SCDM refers to vaccine recommendation under SCDM. *Within each schedule option, multiple strategies (to reflect use of P in place of Q or B, as well as differing dosing intervals) were considered, thus accounting for the total 27 strategies included in the model.

**Table 2.**
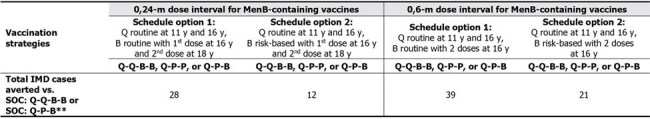
US population total IMD cases averted for vaccination strategies improving PHI versus SOC: Q-Q-B-B and SOC: Q-P-B* B, MenB vaccine; IMD, invasive meningococcal disease; m, months; MenB, IMD caused by meningococcal bacteria serogroup B; P, MenABCWY vaccine; PHI, public health impact; Q, MenACWY vaccine; SOC, standard of care; US, United States; y, years. *Results reflect years 2025–2039, as well as recommendation types for vaccination strategies and coverage assumptions considered in the assessment. **SOC: Q-Q-B-B and SOC: Q-P-B have the same PHI (in terms of cases averted) since clinical characteristics of the vaccines are identical regardless of the comparator.

**Methods:**

A model utilizing epidemiological, vaccination coverage, and economic inputs estimated and compared outcomes for 27 vaccination strategies (using Q, P, and/or B) under schedule options proposed by the ACIP (Table 1) versus (vs) no vaccination (NV) or SOC. Outcomes projected for the US from 2025–2039 included IMD cases, IMD deaths, life-year and quality-adjusted life-year losses due to IMD and IMD-related sequelae, total costs (vaccination and IMD), and incremental CE ratios. A scenario analysis assumed < 100% conversion from SOC (from B to P) in Q-P-B and Q-P-P strategies to consider real-world conversion to P and account for implementation challenges.

**Figure 1.**
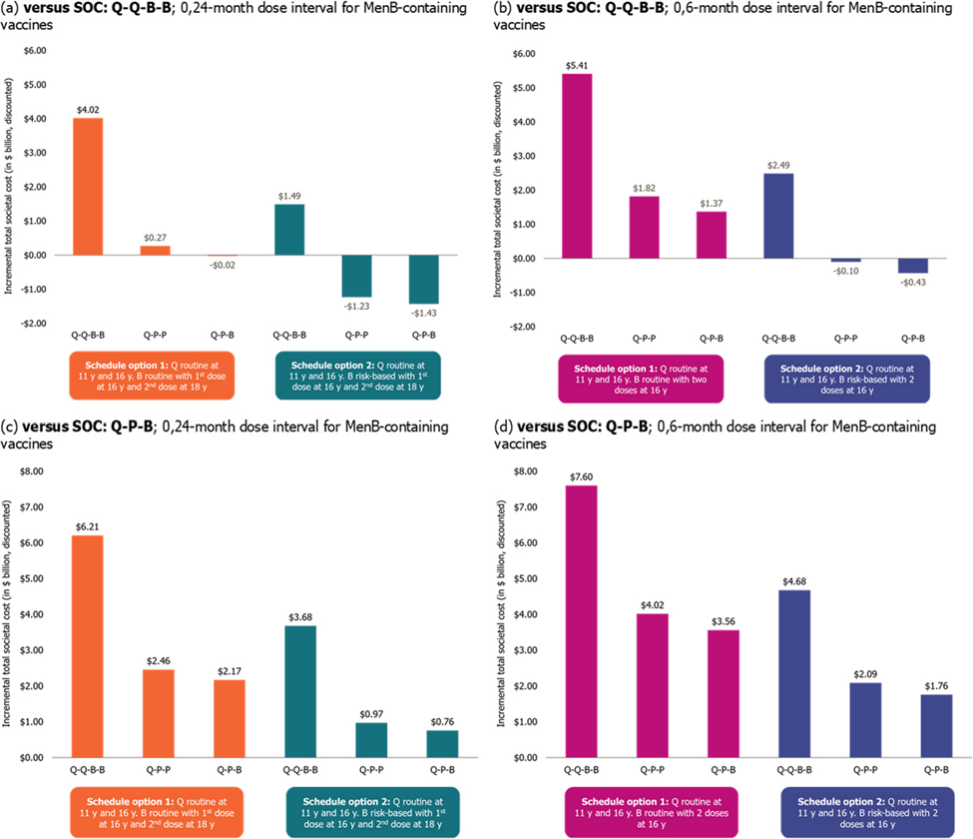
US population incremental total societal cost of vaccination strategies improving PHI versus SOC: Q-B-B and SOC: Q-P-B, years 2025–2039 B, MenB vaccine; IMD, invasive meningococcal disease; MenB, IMD caused by meningococcal bacteria serogroup B; P, MenABCWY vaccine; PHI, public health impact; Q, MenACWY vaccine; SOC, standard of care; US, United States; y, years.

**Results:**

All vaccination strategies improved PHI (i.e., averted IMD cases) vs NV, while those with routine or risk-based MenB-containing vaccines (B or P) at 16 y and routine Q at 11 y improved PHI vs SOC (Table 2). PHI improved the most for strategies using a 0,6-m dosing interval for B or P. Removing Q at 11 y worsened PHI vs SOC. Strategies with routine or risk-based P using 0,6- or 0,24-m dose intervals decreased societal costs vs SOC: Q-Q-B-B (Figure 1). The scenario analysis suggested Q-P-P strategies may be cost saving vs Q-P-B strategies.

**Conclusion:**

Strategies introducing a routine or risk-based recommendation for MenB-containing vaccines at 16 y improved PHI vs each SOC schedule, especially when using 0,6-m dosing intervals, while removing routine Q at 11 y worsened PHI in all relevant scenarios. Incorporating MenABCWY in the adolescent IMD schedule may decrease societal costs vs SOC: Q-Q-B-B.

**Disclosures:**

Oscar Herrera-Restrepo, PhD, GSK: Employee|GSK: Stocks/Bonds (Public Company) Hiral Shah, PhD, GSK: Employee|GSK: Stocks/Bonds (Public Company) Ginita Jutlla, MSc, GSK: Employee|GSK: Stocks/Bonds (Public Company) Jonathan Graham, BSc, GSK: Grant/Research Support Mei Grace, MSc, GSK: Grant/Research Support Justin Carrico, MSc, GSK: Employee|GSK: Stocks/Bonds (Public Company) Zeki Kocaata, PhD, GSK: Employee|GSK: Stocks/Bonds (Public Company) Sara Poston, PharmD, GSK: Employee|GSK: Stocks/Bonds (Public Company) Diana Clements, MD, GSK: Employee|GSK: Stocks/Bonds (Public Company) Anar Andani, BSc, Medical director, GSK: Employee|GSK: Stocks/Bonds (Public Company) Cindy Burman, PharmD, GSK: Employee|GSK: Stocks/Bonds (Public Company)|Pfizer, Inc.: Stocks/Bonds (Public Company)

